# Picture fuzzy Additive Ratio Assessment Method (ARAS) and VIseKriterijumska Optimizacija I Kompromisno Resenje (VIKOR) method for multi-attribute decision problem and their application

**DOI:** 10.1007/s40747-023-01007-5

**Published:** 2023-03-20

**Authors:** Jianping Fan, Dongshuai Han, Meiqin Wu

**Affiliations:** School of Economics and Management, Shanxi University, Taiyuan, 030006 China

**Keywords:** Picture fuzzy set (PFS), ARAS, VIKOR, The correlation coefficient and standard deviation (CCSD) method, Green supplier selection

## Abstract

The purpose of this paper is to study the multi-attribute decision-making problem under the fuzzy picture environment. First, a method to compare the pros and cons of picture fuzzy numbers (PFNs) is introduced in this paper. Second, the correlation coefficient and standard deviation (CCSD) method is used to determine the attribute weight information under the picture fuzzy environment regardless of whether the attribute weight information is partially unknown or completely unknown. Third, the ARAS and VIKOR methods are extended to the picture fuzzy environment, and the proposed PFNs comparison rules are also applied in the PFS-ARAS and PFS-VIKOR methods. Fourth, the problem of green supplier selection in a picture-ambiguous environment is solved by the method proposed in this paper. Finally, the method proposed in this paper is compared with some methods and the results are analyzed.

## Introduction

With the continuous development of modern science, uncertain and vague information is often encountered in the field of decision-making, which is difficult to handle with the traditional accurate numbers. In order to deal with this situation, Zadeh [1] proposed the fuzzy set (FS) theory, which is characterized by the membership degree. In FS theory, membership usually represents a real number and cannot solve the problem of uncertain membership values. Thus, Zadeh [2] proposed the theory of type-2 fuzzy sets on the basis of the FS. Type-2 fuzzy sets can handle the situation of uncertain membership value well. There are many scholars who have studied type-2 fuzzy sets. Tolga [3] proposed the interval-valued Type-2 Gaussian fuzzy sets with finite ranges, and it is combined with the TODIM method to solve a practical problem of medical device selection. Based on the interval type-2 fuzzy, Zhang [4] investigates the fault detection filter design problem for a class of nonhomogeneous higher level Markov jump systems with uncertain transition probabilities.

Then, on basis of the FS, Atanassov [5, 6] further proposed the intuitionistic fuzzy set (IFS) theory, which includes the degree of membership, the degree of non-membership, and the degree of hesitation. Compared with the FS, the IFS can handle more vague and uncertain information. Although the IFS has a huge advantage in dealing with uncertainty and ambiguity, as the reality becomes more and more complex, some situations are also difficult to handle with IFS. Cuong and Kreinovich [7] noticed this situation, an example of voting in real life, the voting results may have four situations: vote for, abstain, vote against, and refusal of the voting. Therefore, Cuong and Kreinovich [7] proposed the picture fuzzy set (PFS). Compared with IFS, PFS can handle more uncertain and fuzzy information.

PFS has become a hot topic of multi-attribute decision-making (MADM) once it was put forward, and it has aroused many scholars to discuss it. First, Cuong and Kreinovich [7] defined some intersection and union operations of PFS, and the formulas of standard Euclidean distance and standard Hamming distance between picture fuzzy numbers (PFNs). Later, to improve the logical operation of PFS, some logical operation operators of PFS are introduced, such as t-norm (Cuong and Pham [8]). For picture fuzzy measures, Wei [9] proposed the picture fuzzy cross entropy and applied it to the MADM. Son [10, 11] proposed a generalized distance measurement for the PFS and extend the basic measure in the PFS to the new measures called the new generalized picture distance measures and picture association measures. To improve the information aggregation operation of PFS, Wei [12] developed some picture fuzzy aggregation operators, such as picture fuzzy weighted average (PFWA) operator, picture fuzzy weighted geometric (PFWG) operator, picture fuzzy ordered weighted average (PFOWA) operator, and picture fuzzy ordered weighted geometric (PFOWG) operator. After that, Wei [13] presented some novel Dice similarity measures of PFS and the generalized Dice similarity measures of the PFS. Also, Wei [14] proposed some novel process to measure the similarity between PFSs and their application. Later, Muhammad [15] introduced a generalized picture fuzzy soft set and applied it in the decision support system, and Zuo [16] introduced the idea of the picture fuzzy graph based on the picture fuzzy relation and proposed some types of picture fuzzy graph. On this basis, Khalil [17] introduced an interval-valued picture fuzzy set and the notion of the interval-valued picture fuzzy soft set theory. Inspired by Pythagorean fuzzy sets (PyFS) (Yager [18]) and q-rung orthopair fuzzy sets (q-ROFS) (Yager [19]), Mahmood [20] extended the PFSs and proposed the spherical fuzzy set (SFS) and T-spherical fuzzy set (T-SFS). Many scholars have proposed some multi-attribute decision-making methods under the fuzzy picture environment to solve some problems, such as Lin [21] combined the MULTIMOORA method with PFSs and used it to solve the problem of site selection of car sharing station and Simic [22] extended the WASPAS method to the PFS environment and applied it to a specific case. Simic [23] extended the combinative distance-based assessment (CODAS) method to the PFS environment and used it to solve the multi-criteria vehicle shredding facility location problem. Arya [24] combined the TODIM with VIKOR and extended it to the PFS environment, and used the entropy method to determine the attribute weight. Si [25] combined the Dempster–Shafer evidence theory, grey relation analysis, and PFS, and used it to solve the COVID-19 medicine selection problem. Jiang [26] proposed a new picture fuzzy multi-attribute group decision-making method based on cumulative prospect theory (CPT) and TODIM, and applied it to food enterprise quality credit evaluation. Many scholars have also extended and supplemented the aggregation operation, similarity measurement, and application scenarios of PFS, such as Mahmood [27] combined the PFS with hesitant fuzzy sets, and proposes some operators such as the picture hesitant fuzzy Bonferroni mean operator. Kamaci [28] proposed some dynamic aggregation operators and Einstein aggregation operators for interval-valued picture hesitant fuzzy sets, and applied them to multi-period decision-making problems. Khan [29] proposed a bi-parameter similarity and distance metric under the PFS environment and applied it in medical diagnosis. Kumar [30] proposed a new picture fuzzy entropy measure and proved that the proposed measure satisfies the axiomatic definition of entropy measures for picture fuzzy sets. Singh [31] proposed some new similarity for PFSs which can distinguish highly similar but different PFSs, and applied it in pattern recognition, cluster analysis, and MADM. Fatma [32] introduced a new hybrid model based on the PFSs and linear assignment, and applied it to a public transport development problem. Ganie [33, 34] proposed a novel picture fuzzy similarity and introduced a new MADM method and introduced an innovative picture fuzzy distance measure. Lu [35] presented a new type of generalized picture fuzzy soft set and applied it to the MADM problems. Tolga [36] evaluated technology selection for three vertical farm alternatives via MCDM methods, and Weighted Euclidean Distance Based Approximation (WEDBA) and Measuring Attractiveness by a Categorical-Based Evaluation Technique (MACBETH) methods were used to evaluate alternatives. Fetanat [37] takes into consideration the applicability of a novel decision support system, namely, a picture fuzzy set (PFS)-based combined compromise solution, and used this method to choose the right technology considering the principles related to sustainability and circularity pillars. Kaya [38] proposed a new picture fuzzy two-stage group decision-making method, and used the proposed method to solve the problem of circular supplier selection. Akram [39] defined an LR flat picture fuzzy number, which is a generalization of trapezoidal picture fuzzy numbers. Almulhim [40] proposed a Multi-criteria Group Decision-Making for prioritizing a set of COVID-19 vaccination alternatives, under a picture fuzzy environment, where the weights for Decisions Experts and criteria are unknown.

Since the beginning of the twenty-first century, with the continuous consumption of fossil energy, the ecological environment has been greatly tested. People began to pay attention to the protection of the ecological environment. Recently, green supply chain management (GSCM) has caused many scholars and managers to study it to reduce the impact on the ecological environment. The green supplier selection (GSS) is the strategic decision of GSCM. From the very beginning, the green supply chain must pay attention to protecting the environment and reducing environmental pollution (Dutta [41]). However, choosing the best green supplier is a big challenge for companies. Khan [42] discussed the GSS problem under the interval-valued q-rung orthopair fuzzy environment. Celik [43] discussed the GSS problem under the environment of interval type-2 fuzzy sets, and solved a specific case with the BWM-TODIM method. Cui [44] analyzed the innovation strategies for the green supply chain management with QFD (quality function deployment) multidimensionally. Kumar [45] applied fuzzy TOPSIS and fuzzy VIKOR to the problem of selecting green suppliers for sponge iron and steel manufacturing. Tian [46] used the TODIM method to solve the problem of green supplier selection under the q-rung orthopair fuzzy set environment. There is still a lack of research on the selection of green suppliers under the picture fuzzy environment.

The current research on PFS mainly proposes some improved multi-attribute decision-making (MADM) methods, which can be applied in many fuzzy and uncertain scenarios. There are also many methods that are used to determine attribute weight information in different situations. However, there are still many problems that cannot be solved by the existing methods, or have some defects. For example, some of the proposed multi-attribute decision-making methods are too complex to apply to practice. Therefore, the improved PFS-ARAS and PFS-VIKOR methods in this paper are proposed to solve some multi-attribute decision-making (MADM) problems.

Compared with the existing literature, the main contributions of this paper are as follows: First, the CCSD method is extended to the picture fuzzy environment and is used to determine attribute weight information. Second, a method to compare the pros and cons of PFNs is introduced. Third, the ARAS and VIKOR methods are extended to the picture fuzzy environment, and the proposed PFNs comparison rules are also applied in the PFS-ARAS and PFS-VIKOR methods. Fourth, the method proposed in this paper is used to solve the problem of green supplier selection in a picture fuzzy environment. Finally, the method proposed in this paper is compared with some methods to illustrate the effectiveness and reliability of the proposed method.

The method proposed in this article has the following advantages: First, the CCSD method is an attribute weight determination method proposed by Wang and Luo [47]. This method calculates the weight information of attributes according to the correlation coefficient and standard deviation of the decision matrix, and can handle the situation where the attribute weight information is completely unknown or partially unknown. This method is very effective in dealing with uncertain information and has broad application prospects. Second, the method proposed in this paper to compare the advantages and disadvantages of PFNs draws on the idea of positive and negative ideal solutions. By comparing the distance between PFNs and positive and negative ideal solutions, the advantages and disadvantages of PFNs are obtained. This method is simple to calculate, and there is no need to calculate the score function and accuracy function of PFNs. Third, the proposed PFS-ARAS method is well suited for quantitative measurements. The criteria for maximum and minimum values are taken into account in the standardization of the decision matrix, and any unit of measurement can be removed. There is a strong compensation ability between standards, and simple calculations do not require complex calculation processes. Finally, the proposed PFS-VIKOR method allows the use of variables with different units of measurement and different types of criteria. The proposed approach ranks the alternatives by taking into account the degree to which each criterion is met.

The rest of the paper is organized as follows: In the section “Preliminaries”, some basic concepts of PFS are introduced. In the section “The correlation coefficient and standard deviation (CCSD) method”, the main idea and main steps of the CCSD method are introduced. In the section “A new method for comparing the pros and cons of PFNs”, a method to compare the pros and cons of picture fuzzy numbers (PFNs) is introduced. In the section “The combination of PFS and some multi-attribute decision-making methods”, the ARAS and VIKOR methods are extended to the picture fuzzy environment, and the proposed PFNs’ comparison rules are also applied in the PFS-ARAS and PFS-VIKOR methods. In the section “The application”, the problem of green supplier selection in a picture fuzzy environment is solved by the method proposed in this paper. In the section “Comparative analysis”, the method proposed in this paper is compared with some methods. In the section “Conclusion”, we make some conclusions of this article.

## Preliminaries

### Definition 1.

For any universal set *X*, the picture fuzzy set (PFS) is of the form (Cuong and Kreinovich [7])

$$ A = \{ (x,\;\alpha (x),\;\gamma (x),\;\beta (x))|x \in X\} , $$

where $\alpha (x)$, $\gamma (x)$, $\beta (x)$: $X \to [0,1][0,1]$ are the membership, the degree of neutral, and the non-membership, respectively, with the condition $0 \le \alpha (x) + \gamma (x) + \beta (x) \le 1$. And the degree of refusal *x* in *X* is the $r(x) = 1 - (\alpha (x) + \gamma (x) + \beta (x))$. The triplet $(\alpha ,\gamma ,\beta )$ is called the picture fuzzy numbers (PFNs).

### Definition 2.

Let A and B be two PFSs in $X = \{ x_{1} ,x_{2} ,...,x_{m} \}$. The normalized picture fuzzy Hamming distances between A and B can be defined as follows (Cuong and Kreinovich [7]):

1 $$\begin{aligned}  d_{H} (A,B) =&\, \frac{1}{m}\sum\limits_{i = 1}^{m} {(|\alpha_{A} (x_{i} ) - \alpha_{B} (x_{i} )| + } |\gamma_{A} (x_{i} )\\ &  - \gamma_{B} (x_{i} )| + |\beta_{A} (x_{i} ) - \beta_{B} (x_{i} )|).\end{aligned} $$

### Definition 3.

Let $a = (\alpha ,\gamma ,\beta )$ be a PFS. The score function and accuracy function can be defined as (Cuong and Kreinovich [7]): $S(a) = \alpha - \beta$, $H(a) = \alpha + \gamma + \beta$.

### Definition 4.

There are two PFNs ($a = (\alpha_{1} ,\gamma_{1} ,\beta_{1} )$ and $b = (\alpha_{2} ,\gamma_{2} ,\beta_{2} )$). We can compare them by the score function and accuracy function (Cuong and Kreinovich [7])

1. If $$S(a) > S(b)$$, then $$a \succ b$$.
2. If $$S(a) = S(b)$$, then:
  If $$H(a) > H(b)$$, then $$a \succ b$$.
  If $$H(a) = H(b)$$, then $$a\sim b$$.

### Definition 5.

Let $a = (\alpha_{1} ,\gamma_{1} ,\beta_{1} )$ and $b = (\alpha_{2} ,\gamma_{2} ,\beta_{2} )$ be two PFNs. The operation rules of PFNs are defined as follows (Wei [12]):

$$ a \oplus b = (\alpha_{1} + \alpha_{2} - \alpha_{1} \alpha_{2} ,\gamma_{1} \gamma_{2} ,\beta_{1} \beta_{2} ) $$

$$ a \otimes b = (\alpha_{1} \alpha_{2} ,\gamma_{1} + \gamma_{2} - \gamma_{1} \gamma_{2} ,\beta_{1} + \beta_{2} - \beta_{1} \beta_{2} ) $$

$$ \lambda a = (1 - (1 - \alpha_{1} )^{\lambda } ,\gamma_{1}^{\lambda } ,\beta_{1}^{\lambda } ). $$

### Definition 6.

Let $a_{j} = (\alpha_{j} ,\gamma_{j} ,\beta_{j} )\;(j = 1,...,n)$ be a collection of PFNs and $w = [w_{1} ,w_{2} ,...,w_{n} ]^{T}$ is the weight vector of $a_{j}$ with the condition $w_{j} > 0$ and $\sum\nolimits_{j = 1}^{n} {w_{j} } = 1$. Then, the picture fuzzy weighted averaging (PFWA) operator is defined as (Wei [12])

2 $$\begin{aligned}& PFWA_{w} (a_{1} ,a_{2} ,...,a_{n} ) = \mathop \oplus \limits_{j = 1}^{n} (w_{j} a_{j} )\\ &\quad = \left( {1 - \prod\limits_{j = 1}^{n} {(1 - \alpha_{{a_{j} }} )^{{w_{j} }} } ,\prod\limits_{j = 1}^{n} {(\gamma_{{a_{j} }} )^{{w_{j} }} } ,\prod\limits_{j = 1}^{n} {(\beta_{{a_{j} }} )^{{w_{j} }} } } \right).\end{aligned}$$

### Definition 7.

[48] Let $a = (\alpha ,\gamma ,\beta )$ be a PFN. A defuzzification method (Son [48]) to obtain a crisp value is

*Step 1*: Distribute the neutral degree to the membership and non-membership degrees as follows:

3 $$ \alpha^{\prime} = \alpha + \frac{\gamma }{2},\quad \beta^{\prime} = \beta + \frac{\gamma }{2}. $$

*Step 2*: Calculate the crisp value by

4 $$ y = \alpha^{\prime} + \frac{{1 + \alpha^{\prime} - \beta^{\prime}}}{2}r,\quad r = 1 - (\alpha + \gamma + \beta ). $$

## The correlation coefficient and standard deviation (CCSD) method

The determination of attribute weight information is a very important part of the multi-attribute decision-making problem. In the decision-making process, if the weight information of the attribute is not known, the decision cannot proceed further, and certain technical means need to be used to determine the weight information of the attribute. The CCSD is the method of determining criteria weight proposed by Wang and Luo [47], concerning standard deviation between the criteria and their correlation coefficients with the global evaluation of alternatives. The CCSD method can be used to determine the criteria weight information when the criteria weight information is completely unknown or partially unknown.

Suppose there is such a multi-attribute decision-making problem. Suppose there are *m* alternatives ($A = \{ a_{1} ,a_{2} ,...,a_{m} \}$) and *n* criteria ($C = \{ c_{1} ,c_{2} ,...,c_{n} \}$). And the weight information of the criteria is represented by $w = [w_{1} ,w_{2} ,...,w_{n} ]^{T}$ with the condition $w_{j} > 0\;(j = 1,...,n)$ and $\sum\nolimits_{j = 1}^{n} {w_{j} } = 1$. The initial decision matrix is denoted by $R = [k_{ij} ]_{m \times n}$ $(i = 1,...,m;\;j = 1,...,n)$ and the $k_{ij}$ represents the evaluation information of alternative *i* under criteria *j*.

The following are the main principles and steps of the CCSD method.

*Step 1*: Obtain the initial decision matrix from relevant experts.

*Step 2*: Normalize the initial decision matrix.

$$ \partial_{ij} = \frac{{k_{ij} - k_{j}^{\min } }}{{k_{j}^{\max } - k_{j}^{\min } }},i = 1,..,m;\quad {\text{for}}\;{\text{benefit}}\;{\text{criteria}} $$

5 $$ \partial_{ij} = \frac{{k_{j}^{\max } - k_{ij} }}{{k_{j}^{\max } - k_{j}^{\min } }},\quad i = 1,..,m;\quad {\text{for}}\;{\text{cost}}\;{\text{criteria}}{.} $$

After normalizing the initial decision matrix, we get the decision matrix $R = [\partial_{ij} ]_{m \times n}$ $(i = 1,...,m;j = 1,...,n)$.

*Step 3*: Calculate the global evaluation value ($\varphi_{i}$) of alternatives under multiple criteria

6 $$ \varphi_{i} = \sum\limits_{j = 1}^{n} {\partial_{ij} w_{j} } ,\quad i = 1,...,m. $$

*Step 4*: Now, we remove criteria $c_{j}$ from the evaluation criteria ($C = \{ c_{1} ,c_{2} ,...,c_{n} \}$) and consider the influence of criteria $c_{j}$ on the whole decision-making process. When criteria $c_{j}$ is removed, the global evaluation value ($\phi_{ij}$) of the alternatives under the remaining evaluation criteria

7 $$ \phi_{ij} = \sum\limits_{h = 1,h \ne j}^{n} {\partial_{ih} w_{h} } ,\quad i = 1,...,m. $$

$\partial_{ih}$ represents the element value in the decision matrix R, and ${w}_{h}$ represents the weight value of the corresponding attribute.

We then consider the correlation coefficient between the removed criteria $c_{j}$ and the global evaluation value of the alternatives

8 $$ \zeta_{j} = \frac{{\sum\nolimits_{i = 1}^{m} {(\partial_{ij} - \overline{\partial }_{j} )(\phi_{ij} - \overline{\varphi }_{j} )} }}{{\sqrt {\sum\nolimits_{i = 1}^{m} {(\partial_{ij} - \overline{\partial }_{j} )^{2} } \cdot \sum\nolimits_{i = 1}^{m} {(\phi_{ij} - \overline{\varphi }_{j} )^{2} } } }},\quad j = 1,...,n $$

9 $$ \overline{\partial }_{j} = \frac{1}{m}\sum\limits_{i = 1}^{m} {\partial_{ij} } ,\quad j = 1,...,n, $$

10 $$ \overline{\varphi }_{j} = \frac{1}{m}\sum\limits_{i = 1}^{m} {\phi_{ij} } ,\quad j = 1,...,n, $$

11 $$ \phi_{ij} = \sum\limits_{h = 1,h \ne j}^{n} {\partial_{ih} w_{h} } ,\quad i = 1,...,m. $$

$\zeta_{j}$ represents the correlation coefficient value. $\partial_{ij}$ represents the element value in the decision matrix R. $\phi_{ij}$ represents the global evaluation value of the alternatives under the remaining evaluation criteria. $\overline{\partial }_{j}$ and $\overline{\varphi }_{j}$ are arithmetic averages.

If the correlation coefficient is closer to 1, it means that criteria $c_{j}$ have little influence on the overall evaluation score of the alternative, and should be given a smaller weight; otherwise, criteria $c_{j}$ should be given a larger weight.

*Step 5*: Based on the previous analysis, we can use the following formula to determine the weight information of attributes:

12 $$ w_{j} = \frac{{\sigma_{j} \sqrt {1 - \zeta_{j} } }}{{\sum\nolimits_{t = 1}^{n} {\sigma_{t} \sqrt {1 - \zeta_{t} } } }},\quad j = 1,...,n. $$

${w}_{j}$ represents the weight value of the corresponding attribute. $\sigma_{j}$ represents the value of the standard deviation of the corresponding attribute, which can be calculated according to Eq. (13).

The $\sigma_{j}$ is the standard deviation (SD) of the values of criteria $c_{j}$ defined by

13 $$ \sigma_{j} = \sqrt {\frac{1}{m}\sum\limits_{i = 1}^{m} {(\partial_{ij} - \overline{\partial }_{j} )^{2} } } ,\quad j = 1,...,n. $$

*Step 6*: Construct a nonlinear optimization model

M-1 $$ \begin{aligned} MinG &= \sum\limits_{j = 1}^{n} {\left( {w_{j} - \frac{{\sigma_{j} \sqrt {1 - \zeta_{j} } }}{{\sum\nolimits_{q = 1}^{n} {\sigma_{q} \sqrt {1 - \zeta_{q} } } }}} \right)}^{2} \\ &\quad s.t.\sum\limits_{j = 1}^{m} {w_{j} = 1,} \;w_{j} \ge 0,\;j = 1,...,m;\;q = 1,...,n. \\ \end{aligned} $$

This nonlinear programming model can be solved by Lingo and MATLAB software. The above nonlinear programming model is under the condition that the criteria weight information is completely unknown. When the criteria weight information is partially unknown, we only need to add the known weight information to the constraints of the above nonlinear programming model to solve. For example, we know that $0.1 < w_{a} < 0.2$, $0.2 < w_{b} < 0.3$. At this time, the constraints of the above nonlinear programming model become: $s.t.\sum\nolimits_{j = 1}^{m} {w_{j} = 1}$, $w_{j} \ge 0$, $0.1 < w_{a}$ $< 0.2$, $0.2 < w_{b} < 0.3$, $j = 1,...,m$.

## A new method for comparing the pros and cons of PFNs

In this section, a method to compare the pros and cons of PFNs is introduced. According to this method, the best and worst of a set of PFNs can be selected. It is well known that PFNs express uncertain and ambiguous information, considering membership, neutrality, and non-membership. Therefore, for any PFNs, the membership degree of 1 is the most ideal situation, which means that the element completely belongs to this domain. The non-membership degree of 1 is the least ideal situation, which means that this element does not belong to this domain at all. The $O^{ + }$ is used to denote the most ideal case ($O^{ + } = (1,0,0)$) and the $O^{ - }$ is used to denote the least ideal case ($O^{ - } = (0,0,1)$). When comparing the pros and cons of a set of PFNs, as long as the compared PFNs are closer to $O^{ + }$, and farther from $O^{ - }$, it means that the PFN is better. Conversely, the farther a PFN is from $O^{ + }$ and the closer it is to $O^{ - }$, the worse the PFN is. For ease of understanding, we can project the three parameters of PFS into a spatial coordinate axis.

**Fig. 1:**
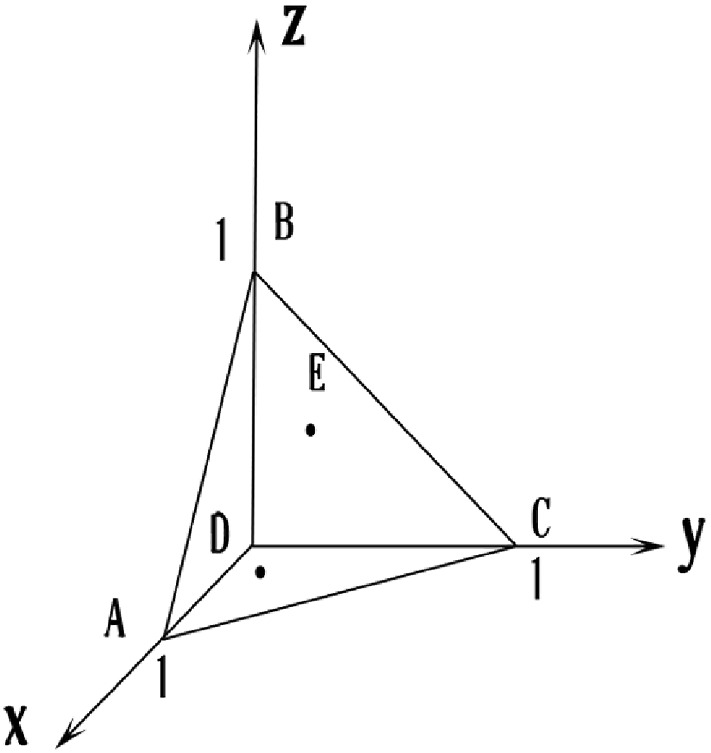
Projection diagram

As shown in Fig. 1, the information represented by membership is projected on the *x*-axis, the information represented by neutrality is projected on the *y*-axis, and the information represented by non-membership is projected on the *z*-axis. Since the value ranges of membership, neutrality, and non-membership are all [0,1], a space composed of points A, B, C, and D is formed. Among them, the most ideal situation is represented by point A, and the least ideal situation is represented by point B. Any PFNs can be projected into this space, as long as it is closer to point A, and farther away from point B, it means that the PFN is better. Conversely, the farther away from point A and the closer to point B, the worse the PFN. In some multi-attribute decision-making methods, the positive and negative ideal solutions need to be obtained from the decision matrix, and we can operate according to this method.

Suppose, there are two PFNs ($a = (\alpha_{1} ,\gamma_{1} ,\beta_{1} )$ and $b = (\alpha_{2} ,\gamma_{2} ,\beta_{2} )$). The $O^{ + }$ is used to denote the most ideal case ($O^{ + } = (1,0,0)$) and the $O^{ - }$ is used to denote the least ideal case ($O^{ - } = (0,0,1)$). PFNs can be compared according to the methods presented in this chapter.

1. If $$d_{ham} (a,O^{ + } ) < d_{ham} (b,O^{ + } )$$, then $$a \succ b$$.
2. If $$d_{ham} (a,O^{ + } ) = d_{ham} (b,O^{ + } )$$, then
  If $$d_{ham} (a,O^{ - } ) > d_{ham} (b,O^{ - } )$$, then $$a \succ b$$.
  If $$d_{ham} (a,O^{ - } ) = d_{ham} (b,O^{ - } )$$, then $$a\sim b$$.

Here, $d_{ham} (a,O^{ + } )$, $d_{ham} (b,O^{ + } )$ denote the standard Hamming distance between PFN ($a,b$) and $O^{ + } = (1,0,0)$, and $d_{ham} (a,O^{ - } )$, $d_{ham} (b,O^{ - } )$ denote the standard Hamming distance between PFN ($a,b$) and $O^{ - } = (0,0,1)$.

For example, there are two PFNS ($\mu_{1} = (0.2,0.3,0.4)$, $\mu_{2} = (0.3,0.3,0.2)$). First, compare the pros and cons of these two PFNs in terms of score function and accuracy function. Because $s(\mu_{1} ) = - 0.2,s(\mu_{2} ) = 0.1$, $s(\mu_{2} ) > s(\mu_{1} )$, so $\mu_{2} \succ \mu_{1}$. In the following, the comparison method proposed in this chapter is used to compare the pros and cons of these two PFNs. Because $d_{ham} (\mu_{1} ,O^{ + } ) = 0.75$, $d_{ham} (\mu_{2} ,O^{ + } ) = 0.6$,$d_{ham} (\mu_{2} ,O^{ + } ) < d_{ham} (\mu_{1} ,O^{ + } )$, so $\mu_{2} \succ \mu_{1}$. This shows that the method proposed in the article to compare PFNs is feasible.

## The combination of PFS and some multi-attribute decision-making methods

### PFS-ARAS

Phase 1: Determine criteria weight.

The CCSD method is used to determine the criteria weight information when the criteria weight information is completely unknown or partially unknown.

*Step 1*: Obtain the initial picture decision matrix $R_{1} = [x_{ij} ]_{m \times n} (i = 1,...,m;j = 1,...,n)$.

*Step 2*: Defuzzification of the initial decision matrix is done according to Definition 7. After the defuzzification process, the decision matrix $R_{2} { = }[k_{ij} ]_{m \times n}$ is obtained.

*Step 3*: Standardize the decision matrix $R_{2}$, the processing rules are as follows. After normalization, the decision matrix $R_{3} = [\partial_{ij} ]_{m \times n}$ is obtained:

$$ \partial_{ij} = \frac{{k_{ij} - k_{j}^{\min } }}{{k_{j}^{\max } - k_{j}^{\min } }},\;i = 1,..,m;\quad {\text{for}}\;{\text{benefit}}\;{\text{criteria}} $$

$$ \partial_{ij} = \frac{{k_{j}^{\max } - k_{ij} }}{{k_{j}^{\max } - k_{j}^{\min } }},\;i = 1,..,m;\quad {\text{for}}\;{\text{cost}}\;{\text{criteria}}{.} $$

*Step 4*: The following nonlinear programming model can be constructed:

$$ MinG = \sum\limits_{j = 1}^{n} {\left( {w_{j} - \frac{{\sigma_{j} \sqrt {1 - \zeta_{j} } }}{{\sum\nolimits_{q = 1}^{n} {\sigma_{q} \sqrt {1 - \zeta_{q} } } }}} \right)}^{2} $$

$$ s.t.\sum\limits_{j = 1}^{m} {w_{j} = 1,} \;w_{j} \ge 0,\;j = 1,...,m;\;q = 1,...,n. $$

According to the above nonlinear programming fuzzy, the weight information of the criteria can be obtained.

Phase 2: Sort alternatives.

*Step 1*: Obtain the initial picture decision matrix $R_{1} = [x_{ij} ]_{m \times n} \;(i = 1,...,m;\;j = 1,...,n)$.

*Step 2*: Normalize the initial picture decision matrix ($R_{1}$). The normalization rules are as follows:

$$ x_{ij} = (\alpha_{ij} ,\gamma_{ij} ,\beta_{ij} );\;{\text{for}}\;{\text{benefit}}\;{\text{attribute}} $$

$$ x_{ij} = x_{ij}^{c} = (\beta_{ij} ,\gamma_{ij} ,\alpha_{ij} );\;{\text{for}}\;{\text{cost}}\;{\text{attribute}}{.} $$

After normalizing the initial picture decision matrix, the picture decision matrix $R_{4} = [r_{ij} ]_{m \times n}$ is obtained.

*Step 3*: A major feature of the ARAS method is to obtain the optimal solution in the decision matrix. The acquisition rules are as follows:

First, calculate the standard Hamming distance between elements $r_{ij}$ and $O^{ + } = (1,0,0)$, and the standard Hamming distance between elements $r_{ij}$ and $O^{ - } = (0,0,1)$. Then, according to the $d_{ham} (r_{ij} ,O^{ + } )$, $d_{ham} (r_{ij} ,O^{ - } )$, the positive ideal solution of the decision matrix can be obtained. Here, the positive ideal solution of the decision matrix is represented by $r_{0j}$. The standard Hamming distance between elements $r_{ij}$ and $O^{ + } = (1,0,0)$ is denoted by $d_{ham} (r_{ij} ,O^{ + } )$, and the standard Hamming distance between elements $r_{ij}$ and $O^{ - } = (0,0,1)$ is denoted by $d_{ham} (r_{ij} ,O^{ - } )$.

$$ r_{0j} = (\alpha_{0j} ,\gamma_{0j} ,\beta_{0j} ) = \{ r_{ij} \;{\text{where}}\;\mathop {\min }\limits_{i} < d_{ham} (r_{ij} ,O^{ + } )|i = 1,...,m\} ;\;j = 1,...,n. $$

*Step 4*: Aggregate the picture fuzzy information ($L_{i}$) under multiple criteria in the alternative (*i*). The evaluation information of each alternative solution will be aggregated into a PFN. The aggregation operation rules are as follows:

14 $$\begin{aligned}& L_{i} = (\alpha_{i} ,\gamma_{i} ,\beta_{i} ) = PFWA_{i} (r_{i1} ,r_{i2} ,...,r_{in} )\\&\quad = \mathop \oplus \limits_{j = 1}^{n} (w_{j} r_{ij} )\\&\quad = \left( {1 - \prod\limits_{j = 1}^{n} {(1 - \alpha_{ij} )^{{w_{j} }} ,\prod\limits_{j = 1}^{n} {(\gamma_{ij} )^{{w_{j} }} } ,\prod\limits_{j = 1}^{n} {(\beta_{ij} )^{{w_{j} }} } } } \right)\quad i = 0,1,...,m. \end{aligned}$$

*Step 5*: Calculate the performance score ($sc_{i}$) of alternative (*i*)

$$ sc_{i} = \alpha_{i} - \beta_{i} ;\quad i = 0,1,...,m. $$

*Step 6*: Calculate the utility value ($k_{i}$) of alternative (*i*)

$$ k_{i} = \frac{{sc_{i} }}{{V_{0} }};\quad i = 1,...,m. $$

$V_{0}$ is the score function value of the positive ideal solution $r_{0j}$.

*Step 7*: Sort them in descending order according to the value of $k_{i}$.

### PFS-VIKOR

Phase 1: Determine attribute weight.

The operation steps here are the same as those in the previous PFS-ARAS, and will not be described here.

Phase 2: Sort alternatives.

*Step 1*: Obtain the initial picture decision matrix ($R_{1} = [x_{ij} ]_{m \times n} (i = 1,...,m;\;j = 1,...,n)$). It is the same as the PFS-ARAS.

*Step 2*: Standardize the decision matrix, and the normalization rules are as follows. It is the same as the PFS-ARAS. After normalizing the initial picture decision matrix, the picture decision matrix $R_{4} = [r_{ij} ]_{m \times n}$ is obtained

$$ x_{ij} = (\alpha_{ij} ,\gamma_{ij} ,\beta_{ij} );\quad {\text{for}}\;{\text{benefit}}\;{\text{attribute}} $$

$$ x_{ij} = x_{ij}^{c} = (\beta_{ij} ,\gamma_{ij} ,\alpha_{ij} );\quad {\text{for}}\;{\text{cost}}\;{\text{attribute}}{.} $$

*Step 3*: The VIKOR method needs to obtain the positive ideal solution and the negative ideal solution from the picture decision matrix ($R_{4} = [r_{ij} ]_{m \times n}$).

First, calculate the standard Hamming distance between elements $r_{ij}$ and $O^{ + } = (1,0,0)$, and the standard Hamming distance between elements $r_{ij}$ and $O^{ - } = (0,0,1)$. Then, according to the $d_{ham} (r_{ij} ,O^{ + } )$ $d_{ham} (r_{ij} ,O^{ - } )$, the positive ideal solution and the negative ideal solution of the decision matrix can be obtained. Here, the positive ideal solution of the decision matrix is represented by $A^{ + }$ and the negative ideal solution of the decision matrix is represented by $A^{ - }$. The standard Hamming distance between elements $r_{ij}$ and $O^{ + } = (1,0,0)$ is denoted by $d_{ham} (r_{ij} ,O^{ + } )$, and the standard Hamming distance between elements $r_{ij}$ and $O^{ - } = (0,0,1)$ is denoted by $d_{ham} (r_{ij} ,O^{ - } )$

$$
A^{ + }  = \left\{ {r_{{ij}} \;{\text{where}}\;\min\nolimits_{i}
\;\langle d_{{ham}} (r_{{ij}} ,O^{ + } )|i = 1,...,m\rangle }
\right\} $$

$$ A^{ - }
= \left\{ {r_{ij} \;{\text{where}}\;\min\nolimits_{i}
\langle d_{ham} (r_{ij} ,O^{ - } )|i = 1,...,m\rangle } \right\}.
$$

$A^{ + }$ can be represented by $r_{j}^{ + } = (\alpha_{j}^{ + } ,\gamma_{j}^{ + } ,\beta_{j}^{ + } )$ and $A^{ - }$ can be represented by $r_{j}^{ - } = (\alpha_{j}^{ - } ,\gamma_{j}^{ - } ,\beta_{j}^{ - } )$.

*Step 4*: VIKOR needs to calculate two initial measures $S_{i}$ and $R_{i}$. The PFS-based measures can be calculated by the following formula:

15 $$ S_{i} = \sum\limits_{j = 1}^{n} {w_{j} \frac{{d_{ham} (r_{ij} ,r_{j}^{ + } )}}{{d_{ham} (r_{j}^{ - } ,r_{j}^{ + } )}}} ,\quad i = 1,...,m,\;j = 1,...,n $$

16 $$ R_{i} = \mathop {max}\limits_{j} \{ w_{j} \frac{{d_{ham} (r_{ij} ,r_{j}^{ + } )}}{{d_{ham} (r_{j}^{ - } ,r_{j}^{ + } )}}\} ,i = 1,...,m;j = 1,...,n. $$

*Step 5*: Calculate the value of $Q_{i}$ can be obtained by the following formula:

17 $$ Q_{i} = v\frac{{S_{i} - S^{ - } }}{{S^{ + } - S^{ - } }} + (1 - v)\frac{{R_{i} - R^{ - } }}{{R^{ + } - R^{ - } }}. $$

Here, $S^{ - } = \mathop {\min }\limits_{i} S_{i}$, $S^{ + } = \mathop {\max }\limits_{i} S_{i}$, $R^{ - } = \mathop {\min }\limits_{i} R_{i}$, $R^{ + } = \mathop {\max }\limits_{i} R_{i}$. $v$ and $1 - v$ are the weight distribution of $S_{i}$ and $R_{i}$, respectively. Here, we take the *v* equal to 0.5.

*Step 6*: Sort them in ascending order according to the values of $S_{i}$, $R_{i}$ and $Q_{i}$. When the following two conditions are met, the compromise solution has the minimum $Q_{i}$ value.

T1. Acceptable advantage: $Q(A_{{{\text{second}}}} ) - Q(A_{{{\text{first}}}} ) \ge \frac{1}{m - 1}$, where $A_{{{\text{second}}}}$ and $A_{{{\text{first}}}}$ are the second position and the first position in the ranking list by $Q_{i}$, and *m* is the number of the alternatives.

T2. Acceptable Stability: The compromise solution must be ranked best in the ranking list of $S_{i}$ and $R_{i}$ values. If either of the two conditions is not met, a set of compromise solutions will be obtained.

- If only T1 is not satisfied, then both $A_{first}$ and $A_{second}$ are compromise solutions.
- If only T2 is not satisfied, then the compromise solution set is:$A_{{{\text{first}}}}$, $A_{{{\text{second}}}}$, …, $A_{L}$, where *L* satisfies the relationship $Q(A_{L} ) - Q(A_{{{\text{first}}}} ) < \frac{1}{m - 1}$. $A_{L}$ is the Lth position in the ranking list by $Q_{i}$.

## The application

With the continuous development of modern society and the continuous consumption of fossil energy, people are facing more and more problems. Among them, the choice of green suppliers is a very important and hot topic. Green supplier selection is essentially a multi-attribute decision-making problem, involving multiple evaluation attributes, and then based on the evaluation of alternative methods under multiple attributes to select alternative solutions. In this part, we apply the improved PFS-ARAS and PFS-VIKOR methods to a green supplier selection problem. Let us consider such a realistic scenario, there are six alternatives ($A_{i} (i = 1,...,6)$) green suppliers. To select the best green suppliers, an expert was invited to evaluate these candidate green suppliers under some evaluation attributes. At present, all countries in the world are paying attention to the deterioration of the earth's environment. Each country has set its own goal of peaking carbon for its own economic development to improve the planet's environment. The automotive industry has also begun to develop in the direction of new energy, and the research and development of traditional gasoline models has gradually weakened. At present, all fields of society are pursuing green and sustainable development. The same goes for supplier selection. Therefore, the resource consumption ($c_{1}$), the carbon emission ($c_{2}$), the green production ($c_{3}$), and the green technology ($c_{4}$) are very important indicators to evaluate the development of green suppliers. These four indicators are very appropriate to evaluate suppliers from the aspects of resource consumption, carbon emissions, green production, and green technology. Obviously, $c_{1}$ and $c_{2}$ are cost attributes, and $c_{3}$ and $c_{4}$ are benefit attributes. We have no information about attribute weights. The picture fuzzy information evaluation matrix given by the expert is shown in Table 1. Next, we use improved PFS-ARAS and PFS-VIKOR methods to deal with this green supplier selection problem.

**Table 1:** The picture fuzzy decision matrix ${R}_{1}$

	${c}_{1}$	${c}_{2}$	${c}_{3}$	${c}_{4}$
${A}_{1}$	< 0.10, 0.29, 0.60 >	< 0.07, 0.27, 0.61 >	< 0.35,0.26,0.27 >	< 0.43,0.32,0.15 >
${A}_{2}$	< 0.09, 0.29, 0.52 >	< 0.22, 0.21, 0.55 >	< 0.43, 0.23, 0.22 >	< 0.21, 0.22, 0.46 >
${A}_{3}$	< 0.34, 0.35, 0.24 >	< 0.71, 0.18, 0.05 >	< 0.27, 0.25, 0.47 >	< 0.19, 0.43, 0.31 >
${A}_{4}$	< 0.23, 0.32, 0.42 >	< 0.26, 0.32, 0.37 >	< 0.47, 0.26, 0.25 >	< 0.32, 0.33, 0.28 >
${A}_{5}$	< 0.19, 0.35, 0.28 >	< 0.46, 0.26, 0.16 >	< 0.30, 0.24, 0.40 >	< 0.28, 0.33, 0.35 >
${A}_{6}$	< 0.43, 0.32, 0.20 >	< 0.34, 0.34, 0.27 >	< 0.45, 0.26, 0.21 >	< 0.44, 0.25, 0.19 >

### PFS-ARAS

Phase 1: Determine attribute weight.

*Step 1*: Defuzzification of the initial decision matrix according to the Definition 7, and the results are shown in Table 2.

**Table 2:** Decision matrix after defuzzification $R_{2} = [k_{ij} ]_{6 \times 4}$

	${c}_{1}$	${c}_{2}$	${c}_{3}$	${c}_{4}$
${A}_{1}$	0.248	0.217	0.545	0.654
${A}_{2}$	0.264	0.332	0.618	0.361
${A}_{3}$	0.554	0.850	0.399	0.436
${A}_{4}$	0.402	0.442	0.612	0.520
${A}_{5}$	0.447	0.668	0.447	0.464
${A}_{6}$	0.621	0.537	0.63	0.640

*Step 2*: Standardize Table 2 according to the following rules, and the results are shown in Table 3:

$$ \partial_{ij} = \frac{{k_{ij} - k_{j}^{\min } }}{{k_{j}^{\max } - k_{j}^{\min } }},\quad i = 1,..,m;\quad {\text{for}}\;{\text{benefit}}\;{\text{criteria}} $$

$$ \partial_{ij} = \frac{{k_{j}^{\max } - k_{ij} }}{{k_{j}^{\max } - k_{j}^{\min } }},\quad i = 1,..,m;\quad {\text{for}}\;{\text{cost}}\;{\text{criteria}}{.} $$

**Table 3:** Standardized decision matrix $R_{3} = [\partial_{ij} ]_{6 \times 4}$

	${c}_{1}$	${c}_{2}$	${c}_{3}$	${c}_{4}$
${A}_{1}$	1	1	0.632	1
${A}_{2}$	0.957	0.818	0.948	0
${A}_{3}$	0.180	0	0	0.256
${A}_{4}$	0.587	0.645	0.922	0.543
${A}_{5}$	0.466	0.288	0.208	0.352
${A}_{6}$	0	0.494	1	0.952

*Step 3*: Construct a nonlinear programming model based on the data in Eq. (5) and Table 3 to obtain attribute weights. The weight information we get by the CCSD method is $w = [0.328,0.07,0.274,0.328]^{T}$.

Phase 2: Sort alternatives.

*Step 1*: The initial picture decision matrix information is shown in Table 1.

*Step 2*: Standardize the initial picture decision matrix in Table 1, and the results are shown in Table 4.

**Table 4:** Standardized decision matrix $R_{4} = [r_{ij} ]_{6 \times 4}$

	${c}_{1}$	${c}_{2}$	${c}_{3}$	${c}_{4}$
${A}_{1}$	< 0.60, 0.29, 0.10 >	< 0.61, 0.27, 0.07 >	< 0.35, 0.26, 0.27 >	< 0.43, 0.32, 0.15 >
${A}_{2}$	< 0.52, 0.29, 0.09 >	< 0.55, 0.21, 0.22 >	< 0.43, 0.23, 0.22 >	< 0.21, 0.22, 0.46 >
${A}_{3}$	< 0.24, 0.35, 0.34 >	< 0.05, 0.18, 0.71 >	< 0.27, 0.25, 0.47 >	< 0.19, 0.43, 0.31 >
${A}_{4}$	< 0.42, 0.32, 0.23 >	< 0.37, 0.32, 0.26 >	< 0.47, 0.26, 0.25 >	< 0.32, 0.33, 0.28 >
${A}_{5}$	< 0.28, 0.35, 0.19 >	< 0.16, 0.26, 0.46 >	< 0.30, 0.24, 0.40 >	< 0.28, 0.33, 0.35 >
${A}_{6}$	< 0.20, 0.32, 0.43 >	< 0.27, 0.34, 0.34 >	< 0.45, 0.26, 0.21 >	< 0.44, 0.25, 0.19 >

*Step 3*: Obtain the positive ideal solution from the decision matrix in Table 4.

Firstly, calculate the Hamming distance between the $R_{4} = [r_{ij} ]_{6 \times 4}$ and (1,0,0), (0,0,1). The results are shown in Tables 5 and 6.

**Table 5:** The Hamming distance between $r_{ij}$ and (1, 0, 0)

	(1, 0, 0)	(1, 0, 0)	(1, 0, 0)	(1, 0, 0)
${r}_{1j}$	0.395	0.365	0.59	0.52
${r}_{2j}$	0.43	0.44	0.51	0.735
${r}_{3j}$	0.725	0.92	0.725	0.775
${r}_{4j}$	0.565	0.605	0.52	0.645
${r}_{5j}$	0.63	0.78	0.67	0.7
${r}_{6j}$	0.775	0.705	0.51	0.5
Min	0.395	0.365	0.51	0.52

**Table 6:** The Hamming distance between $r_{ij}$ and (0, 0, 1)

	(0, 0, 1)	(0, 0, 1)	(0, 0, 1)	(0, 0, 1)
${r}_{1j}$	0.895	0.905	0.67	0.8
${r}_{2j}$	0.86	0.77	0.72	0.485
${r}_{3j}$	0.625	0.26	0.525	0.655
${r}_{4j}$	0.755	0.715	0.74	0.685
${r}_{5j}$	0.72	0.48	0.57	0.63
${r}_{6j}$	0.545	0.635	0.75	0.75
min	0.545	0.26	0.525	0.485

From Table 5, we can know that the minimum values of $r_{i1}$, $r_{i2}$, $r_{i3}$, and $r_{i4}$ are 0.395, 0.365, 0.51, 0.52. The 0.395 corresponds to $r_{11}$, 0.365 corresponds to $r_{12}$, 0.51 corresponds to $r_{23}$ and $r_{63}$, and 0.52 corresponds to $r_{14}$. Because 0.51 corresponds to the two values of $r_{23}$ and $r_{63}$, we then compare the Hamming distance between $r_{23}$, $r_{63}$ and (0,0,1). From Table 6, we can know that the Hamming distances between $r_{23}$, $r_{63}$ and (0,0,1) are 0.72 and 0.75, respectively. Since 0.75 is greater than 0.72, $r_{63}$ is the positive ideal solution of $r_{3j}$. From the above analysis, we can know that the positive ideal solution of Table 4 is: ${r}_{0j}= [(\mathrm{0.60,0.29,0.10}), (\mathrm{0.61,0.27,0.07}), (0.45,\mathrm{ 0.26,0.21}), (\mathrm{0.43,0.32,0.15})]$.

*Step 4*: Aggregate the picture fuzzy information ($L_{i}$) under multiple attributes in the alternative (*i*) according to Eq. (14). The results are shown in Table 7.

**Table 7:** The PFS-ARAS result

	*L*	*sc*	*K*	Rank
${x}_{0j}$	(0.511, 0.289, 0.137)	0.374		
${A}_{1}$	(0.488, 0.289, 0.146)	0.342	0.914	1
${A}_{2}$	(0.410, 0.243, 0.209)	0.201	0.537	2
${A}_{3}$	(0.220, 0.326, 0.380)	−0.160	−0.428	6
${A}_{4}$	(0.400,0.305, 0.253)	0.147	0.393	3
${A}_{5}$	(0.278, 0.303, 0.303)	−0.025	−0.067	5
${A}_{6}$	(0.362, 0.280, 0.266)	0.096	0.257	4

*Step 5*: Calculate the performance score ($sc_{i}$) of alternative (*i*). The results are shown in Table 7.

*Step 6*: Calculate the utility value ($k_{i}$) of alternative (*i*). The results are shown in Table 7.

*Step 7*: Sort them in descending order according to the value of $k_{i}$. The results are shown in Table 7.

From Table 7 we can know that the final alternatives are ranked as follows: $A_{1} \succ A_{2} \succ A_{4} \succ A_{6} \succ A_{5} \succ A_{3}$.

### PFS-VIKOR

Phase 1: Determine attribute weight.

The steps are the same as PFS-ARAS, which will not be described here. The weight information is: $w = [0.328,0.07,0.274,0.328]^{T}$.

Phase 2: Sort alternatives.

*Step 1*: The initial picture decision matrix information is shown in Table 1.

*Step 2*: Standardize the initial picture decision matrix in Table 1, and the results are shown in Table 4 ($R_{4} = [r_{ij} ]_{6 \times 4}$).

*Step 3*: Obtain the positive ideal solution and the negative ideal solution from the decision matrix in Table 4. In the previous PFS-ARAS, we have already obtained the positive ideal solution:$A^{ + } = [(0.60,0.29,0.10),(0.61,0.27,0.07),(0.45,0.26,0.21),(0.43,0.32,0.15)]$. Next, we obtain the negative ideal solution of the decision matrix according to Table 6. From Table 6, we can know that the minimum values of $r_{i1}$, $r_{i2}$, $r_{i3}$, and $r_{i4}$ are: 0.545, 0.26, 0.525, and 0.485. The 0.545 corresponds to $r_{61}$, 0.26 corresponds to $r_{32}$, 0.525 corresponds to $r_{33}$, and 0.485 corresponds to $r_{24}$. From the above analysis, we can know that the negative ideal solution of Table 4 is: $A^{ - } = [(0.20,0.32,0.43),(0.05,0.18,0.71),(0.27,0.25,0.47),(0.21,0.22,0.46)]$.

*Step 4*: Calculate the $S_{i}$ and $R_{i}$ values according to Eq. (15) and Eq. (16), and the results are shown in Table 8.

**Table 8:** The result of the VIKOR method

	${S}_{i}$	${R}_{i}$	${Q}_{i}$	Rank
${A}_{1}$	0.097	0.097	0	1
${A}_{2}$	0.418	0.274	0.584	4
${A}_{3}$	0.894	0.285	0.907	6
${A}_{4}$	0.339	0.147	0.260	2
${A}_{5}$	0.656	0.203	0.580	3
${A}_{6}$	0.427	0.328	0.707	5

*Step 5*: Calculate the value of $Q_{i}$ according to Eq. (17), and the results are shown in Table 8.

*Step 6*: Sort them in ascending order according to the values of $Q_{i}$. The results are in Table 8.

Next, we verify the constraints of the compromise solution.

T1. Acceptable advantage: $0.584 - 0 \ge \frac{1}{m - 1} = \frac{1}{4 - 1} = 0.333$. The compromise solution obtained satisfies this restriction.

T2. Acceptable Stability: It can be seen from Table 8 that $A_{1}$ ranks the best in the $Q_{i}$ value ranking, and is also the best in the $S_{i}$ value and $R_{i}$ value ranking, which meets the limit of acceptable stability.

From Table 8, we can get the alternatives in the PFS-VIKOR method in order as follows: $A_{1} \succ A_{4} \succ A_{5} \succ A_{2} \succ A_{6} \succ A_{3}$. From Table 7, we can get the ranking of the alternatives in the PFS-ARAS method as follows: $A_{1} \succ A_{2} \succ A_{4} \succ A_{6} \succ A_{5} \succ A_{3}$. Comparing the results obtained by the two methods, it is found that the results obtained by the two methods are slightly different. The best and worst alternatives selected by the two methods are the same, while the intermediate alternatives are slightly different. For example, the $A_{4}$ word ranks second in the PFS-ARAS method, and ranks third in the PFS-VIKOR method. However, the best solutions selected by the two are the same, so it can be concluded that these two methods are still effective in the environment of picture fuzzy.

This example shows that the method proposed in this paper has strong application prospects, such as multi-attribute decision-making problems, such as medical diagnosis, pattern recognition, and supplier selection. Through the fuzzy initial decision matrix given by relevant experts, it can be processed according to the method proposed in this paper to obtain the ranking of alternatives. Moreover, the method proposed in this paper is simple to calculate, and the CCSD method can be calculated by writing nonlinear programming models in LINGO, MATLAB, and other software. It can be easily applied to some scenarios.

## Comparative analysis

In this part, some existing multi-attribute decision-making methods are compared and analyzed. The data in Table 1 are the initial picture decision matrix. In this multi-attribute decision problem, there are six alternatives and four evaluation attributes, and $c_{1}$, $c_{2}$ are cost attributes, $c_{3}$, $c_{4}$ are benefit attributes. The weight information of the attribute is $w = [0.328,0.07,0.274,0.328]^{T}$. The results obtained by some of the existing methods are shown in Table 9.

**Table 9:** Comparison of the results of some methods

Method	Sorting of alternatives
PFS-ARAS	$A_{1} \succ A_{2} \succ A_{4} \succ A_{6} \succ A_{5} \succ A_{3}$
PFS-VIKOR	$A_{1} \succ A_{4} \succ A_{5} \succ A_{2} \succ A_{6} \succ A_{3}$
PFS-WASPAS [22]	$A_{1} \succ A_{2} \succ A_{4} \succ A_{6} \succ A_{5} \succ A_{3}$
PFS-EDAS [49]	$A_{2} \succ A_{1} \succ A_{3} \succ A_{6} \succ A_{5} \succ A_{4}$
PFS-COPRAS [50]	$A_{3} \succ A_{1} \succ A_{6} \succ A_{4} \succ A_{2} \succ A_{5}$

From the results in Table 9, it can be known that the ranking of the alternatives calculated by the PFS-ARAS method and the PFS-WASPAS method are completely consistent. The ranking of alternatives calculated by PFS-ARAS, PFS-VIKOR, PFS-EDAS, and PFS-COPRAS methods is not completely consistent. Among them, the results of the best alternative and the worst alternative obtained by the PFS-ARAS, PFS-VIKOR, and PFS-WASPAS methods are the same, which are $A_{1}$ and $A_{3}$, respectively. These methods are slightly different in the ordering of $A_{2}$, $A_{4}$, $A_{5}$, and $A_{6}$. For example, $A_{2}$ ranks second in the PFS-ARAS method, fourth in the PFS-VIKOR method, and second in the WASPAS method. The PFS-EDAS method ranked $A_{2}$ first and $A_{4}$ last. The PFS-COPRAS method ranked $A_{3}$ first and $A_{5}$ last. All of the above methods place $A_{1}$ at the front and $A_{5}$ at the back. From the above analysis, it can be proved that the PFS-ARAS and PFS-VIKOR methods proposed in this paper are effective and reliable.

## Conclusion

The PFS is a very powerful tool in dealing with uncertain and vague information. In this article, a method to compare the pros and cons of PFNs is introduced. Second, the CCSD method is extended to the picture fuzzy environment and is used to determine attribute weight information. The CCSD method can handle the situation where the attribute weight information is completely unknown or partly unknown. Third, the ARAS and VIKOR methods are extended to the picture fuzzy environment, and the proposed PFNs’ comparison rules are also applied in the PFS-ARAS and PFS-VIKOR methods. Fourth, the method proposed in this paper is used to solve the problem of green supplier selection in a picture fuzzy environment. Finally, the method proposed in this paper is compared with some methods to illustrate the effectiveness and reliability of the proposed method.

At the same time, the method proposed in this paper still has some limitations in some aspects. First, the initial fuzzy decision matrix may be difficult to obtain, and in the actual application process, the acquisition of fuzzy evaluation data on alternatives is a difficult point. Second, the attribute weight determination method proposed in this paper needs to defuzz PFNs before calculation, which is still calculated according to the correlation coefficient and standard deviation of the accuracy number, and some fuzzy information may be lost during the defuzzing process.

In future research, with the improvement of PFNs operation rules, the weight value of attributes can be determined directly by calculating the correlation coefficient and standard deviation between PFNs. At the same time, the CCSD method determines that the weight value is calculated according to the data itself, and the subjective factors of the experts can be further considered, and the subjective factors of the experts and the objective factors can be considered to be combined, so as to obtain more reasonable attribute values. There are also interval PF, linguistic term PFS, hesitant PFS, etc. that deserve further study in the future.

## Data Availability

Data sharing not applicable to this article as no datasets were generated or analysed during the current study.
